# Supplementary figures and images for: Metabolomics and Microbiomics Reveal Impacts of Rhizosphere Metabolites on Alfalfa Continuous Cropping
Source: Front Microbiol. 2022 Apr 21;13:833968. doi: 10.3389/fmicb.2022.833968 (PMC9069006; doi:10.3389/fmicb.2022.833968)

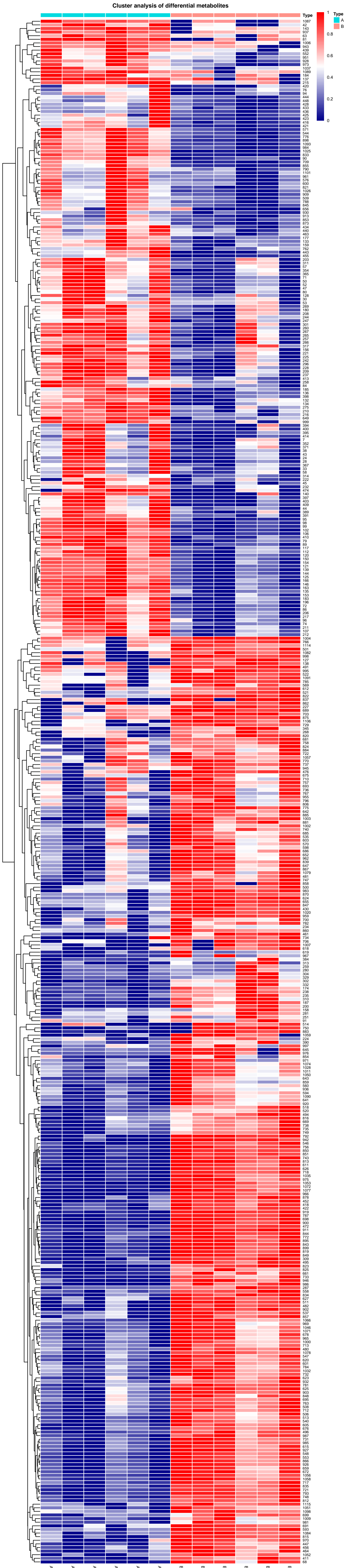

Supplement: Supplementary file 1 [file Data_Sheet_1.pdf]

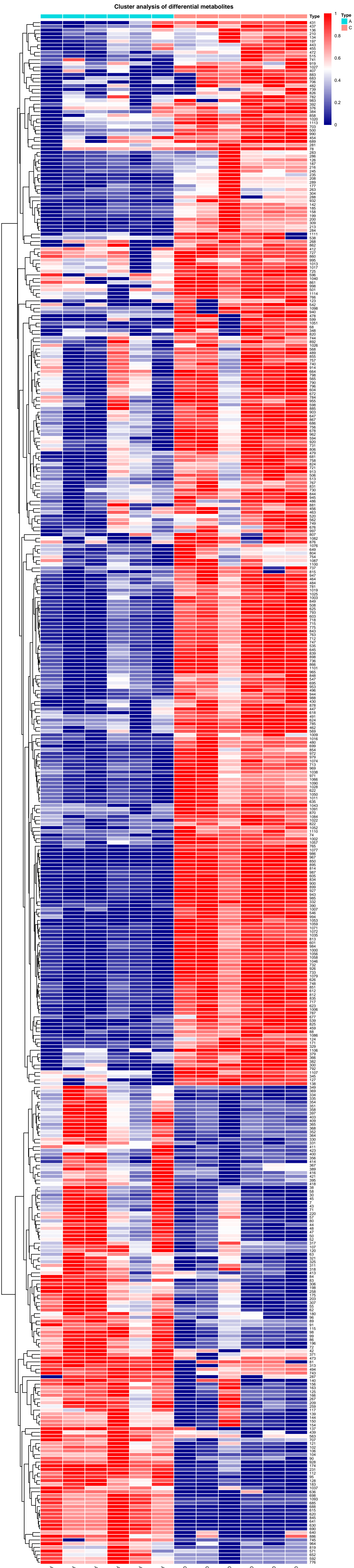

Supplement: Supplementary file 2 [file Data_Sheet_2.pdf]

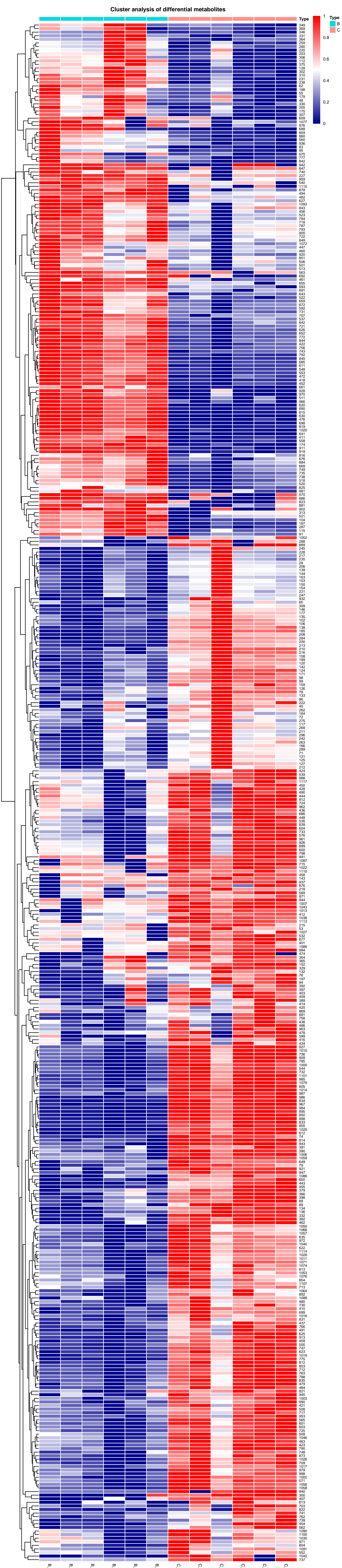

Supplement: Supplementary file 3 [file Data_Sheet_3.pdf]
